# Treatment of Rat Spinal Cord Injury with the Neurotrophic Factor Albumin-Oleic Acid: Translational Application for Paralysis, Spasticity and Pain

**DOI:** 10.1371/journal.pone.0026107

**Published:** 2011-10-26

**Authors:** Gerardo Avila-Martin, Iriana Galan-Arriero, Julio Gómez-Soriano, Julian Taylor

**Affiliations:** 1 Neurología Experimental, Hospital Nacional de Parapléjicos, Servicio de Salud de Castilla-La Mancha, Toledo, Castilla La-Mancha, Spain; 2 Escuela de Enfermería y Fisioterapia de Toledo, Universidad de Castilla La-Mancha, Toledo, Castilla-La Mancha, Spain; Washington University, United States of America

## Abstract

Sensorimotor dysfunction following incomplete spinal cord injury (iSCI) is often characterized by the debilitating symptoms of paralysis, spasticity and pain, which require treatment with novel pleiotropic pharmacological agents. Previous *in vitro* studies suggest that Albumin (Alb) and Oleic Acid (OA) may play a role together as an endogenous neurotrophic factor. Although Alb can promote basic recovery of motor function after iSCI, the therapeutic effect of OA or Alb-OA on a known translational measure of SCI associated with symptoms of spasticity and change in nociception has not been studied. Following T9 spinal contusion injury in Wistar rats, intrathecal treatment with: i) Saline, ii) Alb (0.4 nanomoles), iii) OA (80 nanomoles), iv) Alb-Elaidic acid (0.4/80 nanomoles), or v) Alb-OA (0.4/80 nanomoles) were evaluated on basic motor function, temporal summation of noxious reflex activity, and with a new test of descending modulation of spinal activity below the SCI up to one month after injury. Albumin, OA and Alb-OA treatment inhibited nociceptive Tibialis Anterior (TA) reflex activity. Moreover Alb-OA synergistically promoted early recovery of locomotor activity to 50±10% of control and promoted *de novo* phasic descending inhibition of TA noxious reflex activity to 47±5% following non-invasive electrical conditioning stimulation applied above the iSCI. Spinal L4–L5 immunohistochemistry demonstrated a unique increase in serotonin fibre innervation up to 4.2±1.1 and 2.3±0.3 fold within the dorsal and ventral horn respectively with Alb-OA treatment when compared to uninjured tissue, in addition to a reduction in NR1 NMDA receptor phosphorylation and microglia reactivity. Early recovery of voluntary motor function accompanied with tonic and *de novo* phasic descending inhibition of nociceptive TA flexor reflex activity following Alb-OA treatment, mediated via known endogenous spinal mechanisms of action, suggests a clinical application of this novel neurotrophic factor for the treatment of paralysis, spasticity and pain.

## Introduction

Many patients develop change in sensorimotor function below spinal cord injury (SCI), which include debilitating symptoms of spasticity [1], [2] and changes in pain processing at the spinal level [3]–[5]. These symptoms may combine to impede the successful rehabilitation of residual voluntary motor function following incomplete spinal cord injury (iSCI) [6]. The presence of multiple spinal pathophysiological mechanisms after SCI [7]–[10] require that novel treatments simultaneously modulate neuronal hyperexcitability, neuroinflammation and adaptive or maladaptive neuroplasticity of both segmental and residual descending control systems across the lesion site [9], [11]–[13]. Indeed the increase in neuronal NMDA receptor phosphorylation or glutamate receptor upregulation [14], [15], microglial and astrocyte reactivity [15], [16] and change in descending serotoninergic innervation of spinal activity below the injury [17], [18] have been implicated in symptoms of pain, spasticity and paralysis following iSCI.

Albumin (Alb) is necessary for oleic acid (OA) production by astrocytes [19] and extracellular Alb and OA are incorporated into neurons leading to dendritic growth, GAP-43 and microtubule-associated protein (MAP-2) upregulation *in vitro* [20]. Moreover Alb promotes motor recovery of SCI by neuroprotection against glutamate [21]. While omega-3 fatty acids can promote gain in function [22], no modulation of sensorimotor activity has been observed with omega-9 such as OA following iSCI [22]. However as Alb contains three primary specific binding sites for OA [23], the administration of this serum protein with the omega-9 fatty acid *in vivo,* either individually or in combination, may reveal recovery of unidentified specific sensorimotor function and symptoms following iSCI.

Several clinical studies have identified Tibialis Anterior (TA) hyperreflexia or flexor reflex function as an indirect marker of change in spinal nociceptive function [24]–[26], paralysis [6] and spasticity [26]–[28]. The measurement of cutaneous nociceptive flexor reflex activity in animal models is used routinely to assess pharmacological modulation of spinal excitability [29], [30], including the modulatory role of both tonic and phasic descending modulatory pathways [31]–[34]. Although functional plasticity of descending antinociceptive pathways has not been studied after experimental SCI, their role has been established in phasic inhibition of lumbar cutaneous nociceptive reflex activity following remote electrical conditioning stimulation [34] while dorsolateral funiculus lesion leads to immediate stretch hyperreflexia [35], [36]. As such the evaluation of both tonic and phasic modulation of nociceptive flexor reflex activity may provide important information regarding clinically relevant neuroplasticity within spinal and descending modulatory systems after iSCI [37]–[40], and in this study treatment effects with novel neurotrophic factors such as Alb-OA.

Here we show that local administration of the Alb-OA complex synergistically promoted tonic inhibition of temporal summation of TA noxious reflex activity, *de novo* phasic descending inhibition of nociceptive reflex activity mediated across the iSCI, and early motor recovery [41]. In addition Alb-OA strongly increased lumbar serotoninergic innervation, well above the normal level observed in animals without SCI, reduced phosphorylation of the NR1 NMDA receptor and effectively blocked microglia reactivity. Preclinical development of the Alb-OA complex for the treatment of cutaneous noxious hyperreflexia, altered spinal nociception, spasticity and deficit of voluntary motor function following iSCI will be discussed.

## Materials and Methods

Ten week old male Wistar rats, with an approximate weight of 250–300 g, were used in this study and maintained in the animal resource unit with food and water *ad libitum*, following approval by the institutional animal experimentation ethical committee. The experiments adhered to the guidelines of the Committee for Research and Ethical Issues of IASP published in Pain 1983;16:109–110, following approval from the Ethical Committee for Animal Welfare of the “Hospital Nacional de Parapléjicos” in Toledo (2007; PI07-0806). Animals were subjected to spinal cord injury by moderate T9 contusion. Following SCI, animals were randomly assigned to five groups, each of which was administered with an intrathecal bolus of the following: Saline (Sal, 0.9%, n = 8), Albumin (Alb, 0.4 nanomoles, n = 8), Oleic Acid (OA, 80 nanomoles, n = 8), Albumin-Oleic Acid (Alb-OA, 0.4/80 nanomoles, n = 11) and Albumin-Elaidic Acid (Alb-EA, 0.4/80 nanomoles, n = 11). The compounds were administered in a volume of 10 µl, by the intrathecal route [42], immediately following the SCI and every 3 days thereafter for a total of 28 days. A further group of naïve rats was used as the uninjured control (n = 8), instead of animals with a sham T8 laminectomy because non-significant changes in central sensitization measured by Tibialis Anterior (TA) reflex temporal summation have been identified previously (unpublished data).

### Preparation of the Albumin-Oleic acid complex

The dialysis membrane (D0655-100FT, Sigma-Aldrich, Spain) was prepared by washing it for 4 h under running water under reduced flow. The membrane was then washed, firstly with 0.3% (w/v) solution of sodium sulphide at 80°C for 2 minutes, then with double distilled and deionised water (Milli-Q, Merck) preheated to 60°C for a further 2 minutes, followed with a 0.2% (w/v) solution of sulphuric acid at ambient temperature. Finally, the membrane was washed again with double distilled and deionised water and placed in a receptacle with the same water.

Elliot-Calcium buffer [43] was prepared with the final pH adjusted to 7.4 and filtered with a 0.22 µm pore size (SCGPT05RE, Millipore). Before incubation CaCl_2_.2 H_2_O was added to prepare 1.3 mM Elliot-Ca buffer. Commercially available lyophilised bovine albumin (D0655, Sigma-Aldrich, Spain) was then dialysed by preparing a 10% solution in Elliot-calcium buffer, which was then placed inside the pre-treated dialysis membrane. The dialysis was performed in agitation at 4°C, with 3 washes at 12 hours, 18 and 24 hours respectively. Finally bovine albumin was filtered with a 0.2 µm pore size (4525, Serum Acrodisc® 37 mm syringe filter, Pall Gelman Laboratory). Aliquots of 1 ml bovine albumin were prepared and stored at −20°C.

The albumin-oleic acid complex was prepared with 2% (w/v) bovine albumin solution, by adding oleic acid (O1008, Sigma-Aldrich, Spain) [20]. An albumin-oleic acid (1∶1) solution was prepared to a concentration of 80 nanomoles in saline (0.9%). The albumin-elaidic acid complex was similarly prepared to the same concentration (E4637, Sigma-Aldrich, Spain).

### Experimental animal surgery

Wistar rats were anesthetized with pentobarbital (Dolethal, Vétoquinol, 65 mg/kg, i.p., Ref: 737) and xylazine (Xilagesic 10 mg/kg concentration, i.p. Calier, Ref: 25225), followed by a supplementary 30% dose following aproximately 90 minutes of experimental surgery. In addition 0.1 ml of antibiotic was administered (2.5% Baytril, Enrofloxacin, Bayer) after surgery, followed by daily doses up to 3 days after spinal cord injury.

Commercially available rat intrathecal catheters (ALZT7740Z, Charles River Laboratories, Spain) were implanted (see below) and externalized accordingly [42]. Immediately before surgical implantation, the catheter was re-sterilised with absolute ethanol, and thoroughly washed with sterile 0.9% saline. Following skin incision and blunt dissection of the muscle layers overlying the vertebrae, a small hemi-laminectomy at the vertebral T10 level was performed (spinal T9). The exposed T9 dura was subjected to a small durectomy with iris-type scissors so that the tip of the intrathecal catheter could be inserted rostrally and medially on top of the spinal cord with a final position just below the intended T9 contusion site. The area was cleaned to permit catheter fixture with acrylate cement to the T11 vertebrae. The percutaneous end of the intrathecal catheter was finally secured by inserting it through a small cutaneous incision at the base of the cranium, whereupon it was filled with 0.9% sterile saline and tapped with a custom-made nylon filament.

Following intrathecal catheter implantation a spinal T9 contusion was performed [44]. A bilateral T9 vertebral laminectomy enabled spinal contusion by allowing an 11-gram weight to fall from a height of 12 mm onto a cylindrical flat-tipped impactor with a 2.5 mm diameter placed centrally over the exposed spinal cord above the intact dura. Once the contusion was performed, artificial dura mater (Neuropatch, Ref: 106403, B. Braun), the overlying muscle layers were reapposed with a continuous suture stitch and the skin was finally closed with a subdermal suture, both with a 4-0 reabsorbable thread. Rats were carefully observed during recovery, and the bladder was manually expressed daily until recovery of function.

### Behavioural analysis of evoked voluntary motor function

Evoked voluntary motor function before and after T9 contusion injury was analyzed in all experimental groups with the Rotarod test (4600, Ugo Basile), instead of the standard BBB test [45], because of the specific need to document early the maximal evoked capacity for generalised hindlimb-forelimb locomotor function after SCI. Prior to contusion injury, each animal was trained for three days to remain upon a cylindrical surface which rotated at 5 rpm for at least 5 minutes [46]. On the day before SCI control data were obtained by subjecting the rats to the Rotarod test, but with the cylinder rotating at a steadily accelerating speed from 5 to 15 rpm during the 5 minute test duration [46]. Following SCI rats were tested on day 4 and then weekly thereafter up to 28 days to document general motor recovery and the effect of the different treatments.

### Nociceptive Tibialis Anterior reflex activity and phasic descending inhibition

Four weeks after spinal cord injury, the rats were rapidly anesthetised with isoflurane (2%) in medicinal air (17% oxygen, at 2 l/min, Synthetic medical air, Ref: X505, Carburos Metallicos) for neurophysiological analysis of nociceptive Tibialis Anterior spinal reflex activity. The nose was then inserted into a plexiglass adapter (Cibertec, S.A.) so that the isoflurane-air mixture (2.0%, 1 l/min) could be administered directly, followed by the subcutaneous injection of atropine. The animal was placed in a supine position on an electric blanket maintained at 37°C (RTC1 Thermal Regulator, Cibertec S.A.). Hair over the left TA muscle and at the mid-thoracic level was removed and both the trunk and the hindlimbs were extended and fixed into a neutral position with adhesive tape during the entire experiment. Bipolar electromyographic responses were recorded by subcutaneaously inserting two multi-stranded Teflon®-coated steel electrodes (Cooner Wire, USA) 0.5 cm into the belly of the Tibialis anterior (TA) muscle of the left limb. In addition, two platinum subdermal electrodes (Ref. E2, Astro-Med Inc., Grass Instruments, USA) were inserted into the tip of the fourth toe and secured with adhesive tape. Lastly, an earth electrode was inserted subcutaneously between the stimulation electrode and the recording electrode at the level of the left ankle.

Prior to beginning reflex EMG measurements, the isoflurane anaesthesia level was lowered to 1.2% MAC in medicinal air (1 l/min). The evoked electromyographic responses of the TA muscle were recorded at a gain of 2K, with a bandwidth filter of between 30 Hz – 10 KHz using a differential AC amplifier (AE3, Cibertec S.A.). The electrical stimulus was applied via a stimulation isolation unit (ISU 165, Cibertec S.A.) from an analogue stimulator (CS20BP, Cibertec S.A.), presented as pulses of 2.0 ms duration. Reflex threshold was identified by characterising the minimal current intensity (mA) required to evoke a clear nociceptive TA reflex EMG response between 0.2 and 1.0 s after stimulation, in over half of ten stimuli. Nociceptive TA reflex activity and temporal summation was evoked during a train of 16 stimuli applied at 1 Hz.

The role of descending inhibitory pathways across the T9 spinal cord injury upon nociceptive TA reflex activity was assessed indirectly by applying a percutaneous high frequency electrical conditioning stimulus across the T6-7 vertebral level, by applying an electrical stimulation protocol that has previously been employed to modulate C-fibre evoked potentials [47]. Two platinum subdermal needle electrodes (Ref: E2, Astro-Med INC. Grass, USA) were inserted into each side of the trunk at the T6-7 vertebral level, in order to deliver a stimulus just above the level of the T9 experimental contusion. The high frequency conditioning protocol consisted of a 1 s 100 Hz train, with pulse duration of 0.1 ms, applied every 10 s during a total period of 15 minutes. One minute immediately following the mid-thoracic electrical conditioning stimulation, nociceptive TA reflex activity was recorded using the protocol described above. Similar conditioning electrical stimulation applied to forelimb nerves have been shown to mediate long-lasting inhibition of nociceptive cutaneous spinal reflex activity via a noradrenergic sensitive mechanism [34].

Data were collected with an analog-digital converter (CED Micro 1401 MkII A/D, CED, UK) with data collected at 20 KHz sampling rate. Electromyographic data were integrated using the modulus function of the analysis software (Spike 2, CED, UK) between 0.2 and 0.6 s after the stimulus. Integrated reflex EMG data were analyzed after each stimulus and also grouped for the first and second phase of temporal summation in light of published evidence suggesting that modulation of spinal excitability via descending pathways is evident after a few stimuli [31].

### T9 SCI contusion volume analysis and lumbar L4–L5 immunohistochemistry

At 30 days after SCI, animals were euthanized with sodium pentobarbital followed by intracardiac perfusion with saline and 4% paraformaldehyde in 0.1 M phosphate buffer (PB). The extracted spinal tissue was transferred to 30% sucrose in 0.1 M phosphate buffer and kept at 4°C for at least two days to provide cryoprotection.

Both T6-T13 and lumbar L4–L5 tissue were cut at 30 µm in a Leica CM1900 cryostat and preserved at −30°C until use. Serial sections were collected on slides at a distance of 210 µm between consecutive sections. Thoracic tissue were prepared with Van Gieson's stain and the volume of the spared tissue was calculated with Cavalieri's method for volume estimation (see below). For immunohistochemical analysis sections were preincubated for 1 h in a blocking solution composed of 5% normal goat serum (NGS, Vector) and 0.2% Triton X-100 (Merck) diluted in 0.1 M PB in saline. After the general blocking steps, sections were incubated overnight at 4°C with rabbit primary antibodies against serotonin (rabbit anti-5HT, 1∶4000, Ref: S5545, Sigma-Aldrich) or microglia (Mouse, anti-CD11b clone OX-42, 1∶5000, Ref: Ab1211-100, Abcam). Blocking for nonspecific background staining for pNR1 expression analysis was performed by incubating with a protein block serum (Ultra-V-Block, Ref. TA-125-UV, Thermo Scientific) for 5 minutes at room temperature, prior to overnight incubation with the primary antibody at 4°C (rabbit anti-pNR1, Ser 897 phosphorylated, 1∶1000 Ref: DAM1475052; Millipore).

Following primary antibody incubation, the tissue was washed three times in 1% NGS (except for pNR1 where PBS was used) and then incubated with the appropriate secondary antibody conjugated with Alexa Fluor 594 (1∶1000; 1 hour 4°C; anti-rabbit, Ref: A11012, Invitrogen) or Alexa Fluor 488 (1∶1000; 1 hour 4°C; anti-mouse, Ref: A11012, Invitrogen). The sections were then mounted with Fluoromount (Ref F4680, Sigma-Aldrich) and coverslips for the preservation of fluorescence-stained slide tissue (Thermo Scientific Superfrost Ultra Plus, Ref: J3800AMNZ) and analyzed with a fluorescent microscopy.

### Image acquisition and analysis

Spinal cord volume of the injured region was calculated by Cavallieri's method for volume estimation [48] using Newcast image analysis software (Visiopharm Integrator Software, Visiopharm). Images of injured and uninjured spinal cords were visualized with VIS in an Olympus BX61 microscope with a DP71 digital camera. Data are expressed as mm^3^ of tissue referred to a 1 mm slice of spinal cord.

For immunohistochemical spinal cord tissue analysis, digital images of the tissue were obtained with a confocal microscope (Leica SP5, Leica) and processed with NIH Image J software (Image J 1.38x, NIH). For each antibody, measurements were carried out over 10–20 images (minimum of 3 images/section/animal; n = 6–7 rats per group) which were sampled from digital images of the tissue obtained with a Leica DFC350 FX camera attached to a DM5000 B Leica epifluorescence microscope. Microscope illumination and data acquisition settings were fixed throughout the entire analysis procedure for each specific analysis.

To maintain a constant detection threshold for each image and to compensate for subtle variability of the immunostaining procedure between sections, only immunoreactive 5HT innervation density and number of cells that expressed pNR1 were analysed if the image was at least 50% darker than the average grey level after background subtraction, and shading correction were performed in the areas of grey matter. General 5-HT analysis was performed for the dorsal and ventral horn, while OX-42 area and number of pNR1-positive cells were analyzed specifically within laminae I-II and the IV–VI (with the exception of pNR1 analysis which was performed in lamina V). The OX-42 analysis protocol was performed as previously published [49]. The immunoreactive 5-HT and OX-42 density in addition to the number of cells expessing pNR1 were averaged and presented as group data. The quantification was presented for 5-HT innervation and OX-42 density as the area in µm^2^, in addition to the number of cells which positively expressed p-NR1 within the dorsal horn. All analytical procedures were performed blind, without knowledge of the experimental conditions.

### Statistical tests

Data were analyzed using a one-way ANOVA for comparisons between groups, while a two-way ANOVA was used for longitudinal analysis such as with the rotarod tests, or for analysis of the temporal summation of TA nociceptive reflex activity. Post-hoc tests were performed with the Students t test (rotarod analysis), the Tukey post-hoc test (electrophysiological data) and the Bonferroni test (immunohistochemistry, Graphpad Prism 4.00, USA).

## Results

All experimental groups demonstrated an increase in body weight during the 28 day study following moderate T9 contusion SCI, suggesting that none of the pharmaceutical treatments were associated with poor health and or adverse drug reactions. Animals treated with saline revealed an increase of 22.9±5.6% body weight during the one month SCI period, while albumin alone (49.9±4.8%) and oleic acid alone (48.6±2.5%) applied via the intrathecal route significantly potentiated weight gain compared to saline alone (p<0.05). Animals treated with albumin-oleic acid also revealed an increase in body weight (39.9±4.6%).

### Synergistic early recovery of evoked motor function after iSCI with Alb-OA

**Figure 1:**
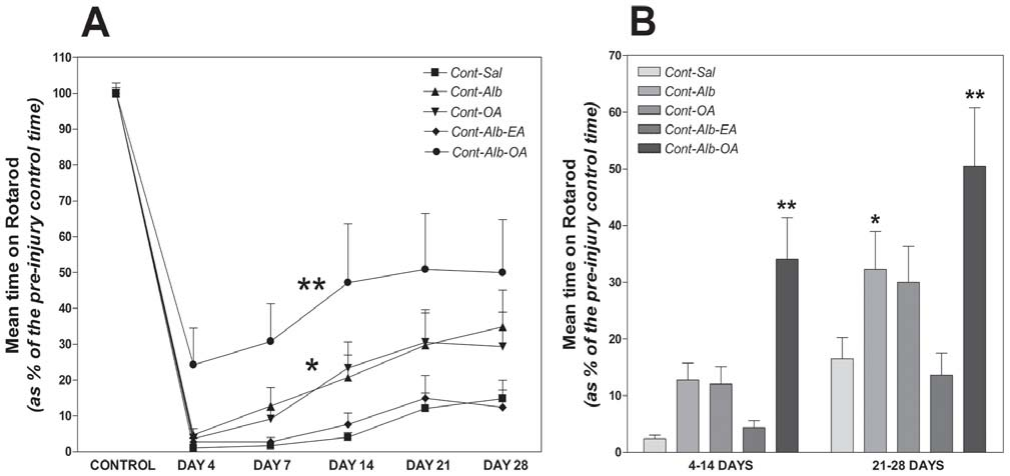
Albumin-Oleic Acid (Alb-OA) Synergistically Promotes Early Recovery of Motor Function following T9 Spinal Cord Injury. Longitudinal analysis of the mean time spent on the rotarod following contusion (Cont) SCI from 4 to 28 days revealed that intrathecal administration of both Alb (0.4 nanomoles, p<0.05) and Alb-OA (80 nanomoles, p<0.01) promoted better motor recovery, compared to the spinal contusion group treated with saline (Sal) alone (Fig. 1A). Treatment with OA (80 nanomoles) alone or Alb-Elaidic acid (Alb-EA, 0.4/80 nanomoles) failed to potentiate locomotor recovery (Fig. 1A). Analysis of voluntary motor function during the early (4–14 day) and subacute (21–28 day) period of contusion SCI demonstrated that Alb-OA (0.4/80 nanomoles) synergistically promoted functional recovery soon after contusion injury when compared to animals treated with saline (Sal) alone, while Alb treatment enhanced the time spent on the rotarod specifically during the late phase (Fig. 1B). Statistical analysis was performed with a two-way (Fig. 1A) and one-way ANOVA (Fig. 1B), supported by a post-hoc Bonferronni test for each experimental group compared with animals with SCI treated with Sal alone (* - p<0.05; ** - p<0.01).

Four days after T9 contusion SCI in animals treated with saline evoked voluntary motor function as assessed on the rotarod was reduced to 1.0±0.1% when compared to the pre-lesion control value (100.0±2.4%, Fig. 1A). Two-way repeated measures ANOVA revealed a difference in recovery rate of voluntary motor function between all experimental groups with different treatments (F = 17.7; d.f. = 4,3; p<0.001). The experimental SCI group treated with albumin-oleic acid demonstrated the greatest recovery of voluntary movement to 50.0±14.8% at 28 days compared to the saline-treated control group (F = 8.96, d.f. = 1, p<0.01). However animals treated with albumin alone also revealed recovery of motor function up to 28 days after SCI to 34.9±10.3%, when compared to the control group (F = 5.6, d.f. = 1, p<0.05).

The effect of albumin-oleic acid upon evoked voluntary motor function was also evident during acute SCI (Fig. 1B). When all data were analyzed between 4–14 and 21–28 days after SCI an early synergistic effect of albumin-oleic acid on motor function recovery was evident. Specifically, motor function measured from 4–14 days after SCI with saline treatment was reduced to 2.4±0.6%, but was significantly potentiated to 34±7% following albumin-oleic acid treatment (Fig. 1B, unpaired two-tailed t-test, t = 4.63; d.f. = 4; p<0.01). Treatment with albumin or oleic acid alone during the acute phase of SCI failed to promote significant motor recovery. The early effect of albumin-oleic acid treatment was maintained during the sub-acute phase of SCI from 21 to 28 days (Fig. 1B). Specifically animals treated with albumin-oleic acid recovered up to 50±10% of the pre-lesion control level of motor activity compared to the saline-treated group (t = 25.8; d.f. = 2; p<0.01), while the group treated with albumin also revealed a modest recovery to 32±7% (t = 6.5; d.f. = 2; p<0.05).

### Tonic inhibition of TA reflex activity with Alb, OA and Alb-OA treatment after iSCI

**Figure 2:**
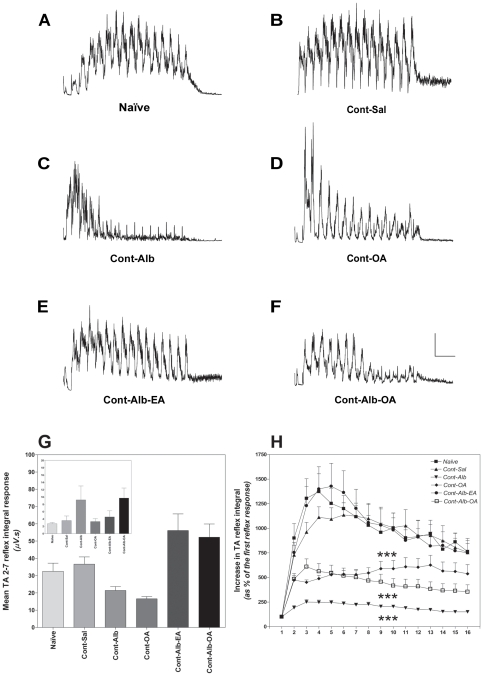
Inhibition of Tibialis Anterior Reflex Activity with Albumin, Oleic Acid and Albumin-Oleic Acid after SCI. Qualitative analysis of rectified nociceptive Tibialis Anterior (TA) noxious reflex temporal summation at 28 days following spinal contusion (Cont, Fig. 2A–2F, scale bar 20 µV/2.5 s) supported the quantification of reflex inhibition (Fig. 2H) with either albumin (Alb, 0.4 nanomoles, p<0.001), oleic acid (OA, 80 nanomoles, p<0.001) or the complex Alb-OA (0.4/80 nanomoles, p<0.001) when compared to animals with SCI treated with saline alone (Sal). Importantly noxious TA reflex activity following moderate T9 contusion injury was not different for either the averaged response calculated from the second to seventh stimulus (Fig. 2G) or the first reflex response (Fig. 2G inset, same format) amongst the experimental treatment groups when compared to the responses observed in naïve animals or with SCI treated with saline alone. Although no increase in temporal summation for the first to the sixteenth reflex response was identified following contusion SCI (Fig. 2H), significant inhibition of noxious TA temporal summation in animals with contusion SCI was observed with albumin (p<0.001), oleic acid (OA, p<0.001) and albumin-oleic acid (Alb-OA, p<.001) when compared to the group treated with saline alone (Sal, Fig. 2H). The stereoisomer complex albumin-elaidic acid (Alb-EA, 0.4/80 nanomoles) failed to inhibit flexor reflex activity following contusion SCI (Fig. 2H). Statistical analysis was performed with a two-way ANOVA supported by the post-hoc Bonferronni test compared to the SCI group treated with saline alone (*** - p<0.001).

Rectified TA reflex EMG activity recorded in response to 16 noxious electrical stimuli was present in naïve (Fig. 2A) and animals with experimental T9 contusion SCI with different treatments (Fig. 2B–2F), illustrating a strong inhibitory effect with albumin (Fig. 2C) or Alb-OA alone (Fig. 2F) on temporal summation. Measurement of absolute TA noxious reflex activity amongst the different groups in response to the first stimulus (inset Fig. 2G) or averaged over the second to seventh response (Fig. 2G) revealed no treatment effect on general activity level, further supporting the use of temporal summation as a measure of change in spinal central sensitisation to noxious stimuli. In animals with SCI treated with saline alone the temporal summation of the nociceptive TA flexor reflex was observed up to a maximal value of 1136±326% (average of 921±85% during the 16 stimuli) when compared to the first reflex response (Fig. 2H). In general temporal summation of the TA noxious flexor reflex was modulated differently between the experimental groups (F = 42.69; d.f. = 4,5; p<0.001). A similar level of maximal TA temporal summation was observed in naïve rats (1304±564%, Fig. 2H). Post-hoc analysis revealed that temporal summation of the TA nociceptive reflex was inhibited following albumin (195±13%; Tukey post-hoc test, p<0.001, q = 14.36), oleic acid alone (521±44%; Tukey post-hoc test, p<0.001, q = 7.91) or albumin-oleic acid (434±46%; Tukey post-hoc test, p<0.001, q = 9.62), when compared to the SCI control group treated with saline alone (921±85%, Fig. 2G). Interestingly animals treated with albumin alone displayed greater inhibition of TA nociceptive reflex temporal summation compared to those treated with albumin-oleic acid (Tukey p<0.05, q = 4.7).

When TA nociceptive reflex data were analyzed during the early (2–7^th^) or late (8–16^th^) response (data not shown), intrathecal administration of albumin alone, oleic acid alone and albumin-oleic acid were shown to inhibit both the first and second phase of temporal summation 28 days following contusion SCI when compared to the group treated with saline alone (first phase: 1026±117%; second phase: 941±125%). Specifically the first phase of TA temporal summation was inhibited by Alb (232±24%, t = 6.56, d.f. = 94, p<0.001), OA (501±40%, t = 4.03, d.f. = 88, p<0.001) and Alb-OA (534±79%, t = 3.61, d.f. = 112, p<0.001) when compared to the saline treated group with SCI. Similarly the second phase of TA temporal summation was reduced by albumin (181±14%, t = 6.56, d.f. = 94, p<0.001), oleic acid (581±70%, t = 6.56, d.f. = 133, p<0.05) and albumin-oleic acid (403±62%, t = 4.18, d.f. = 169, p<0.001).

### Synergistic de novo phasic inhibition of TA reflex activity across the iSCI with Alb-OA

**Figure 3:**
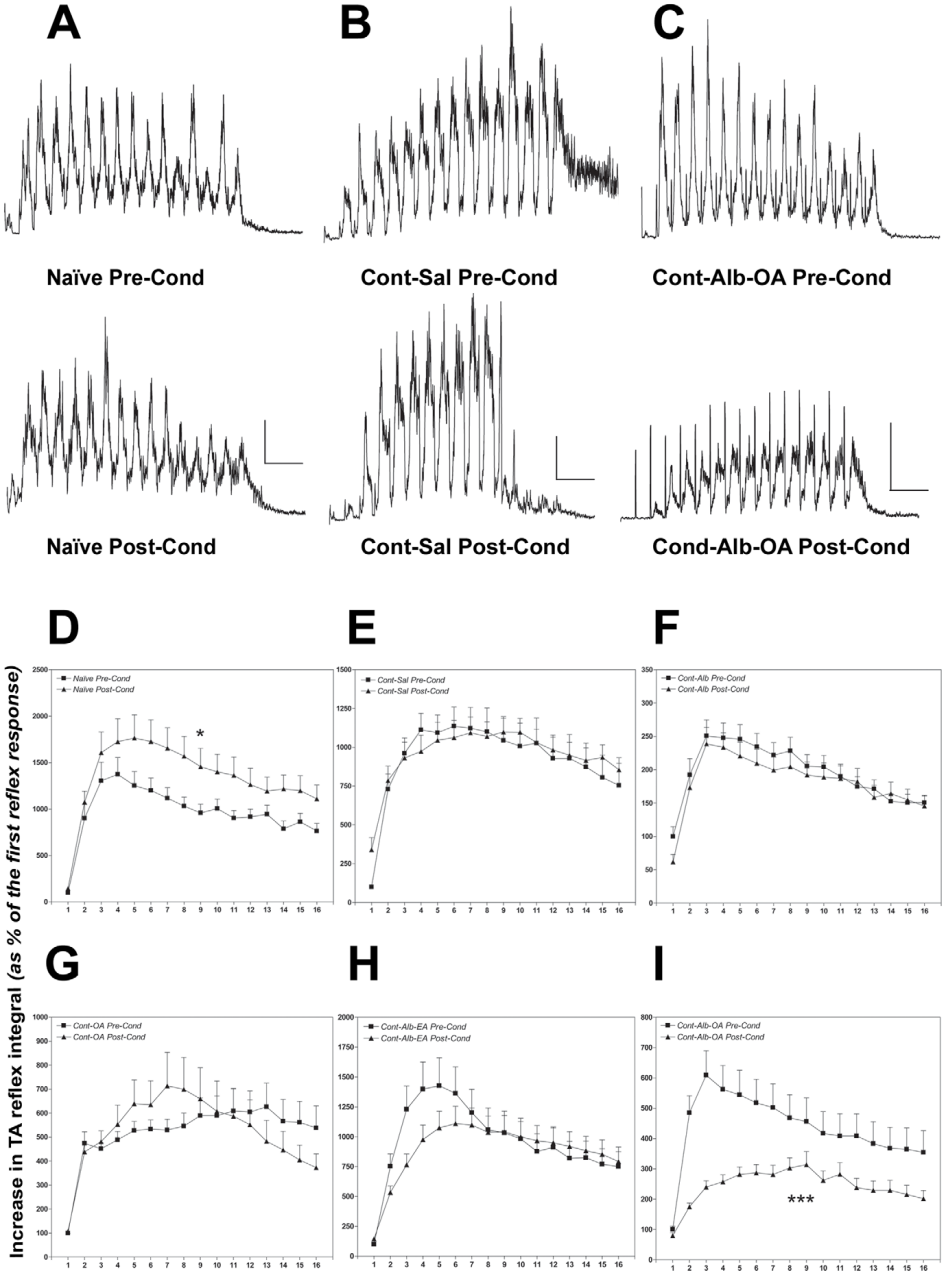
Albumin-Oleic Acid Promotes *De Novo* Synergistic Descending Inhibition of Tibialis Anterior Reflex Activity after SCI. Examination of rectified Tibialis Anterior (TA) electromyograhic reflex activity (Fig. 3A–3C) revealed strong inhibition of temporal summation in animals with T9 spinal contusion (Cont) treated with albumin-oleic acid (Alb-OA, 0.4/80 nanomoles) following high-frequency conditioning stimulation (Post-Cond) above the SCI when compared to the pre-conditioning (Pre-Cond) response (Fig. 3C, scale bar 10 µV/2.5 s). No inhibition was observed either in naïve (Fig. 3A, scale bar 20 µV/2.5 s) or in animals with contusion SCI treated with saline alone (Fig. 3B, scale bar 20 µV/2.5 s). Quantitative analysis of TA reflex temporal summation (Fig. 3D–3I) demonstrated a phasic facilitation in naïve animals following high-frequency electrical conditioning (Fig. 3D), which was absent in the experimental SCI groups (Fig. 3E–3I). Indeed *de novo* synergistic phasic inhibition of TA reflex activity mediated across the SCI was only promoted by treatment with albumin-oleic acid (Alb-OA, Fig. 3I, p<0.001), but not with albumin (Alb, Fig. 3F), oleic acid (OA, Fig. 3G) or albumin-elaidic acid (Alb-EA, Fig. 3H). Statistical analysis was performed with a one-way ANOVA for each experimental group (Fig. 3D–3I, * - p<0.05; *** - p<0.001).

Percutaneous high-frequency electrical conditioning stimulation applied across the spinal cord above the contusion T9 SCI revealed a strong phasic inhibition of nociceptive TA reflex temporal summation following albumin-oleic acid treatment in animals with SCI. Examination of the rectified TA reflex activity recorded from animals with SCI and treated with albumin-oleic acid before (Pre-Cond, Fig. 3C) and after (Post-Cond, Fig. 3C) high-frequency electrical conditioning stimulation revealed this strong inhibition of temporal summation (Fig. 3I). Synergistic phasic inhibition of TA nociceptive reflex activity was evident in animals treated with albumin-oleic acid from a peak of 609±212% to 314±115% (p<0.001, Fig. 3I) compared to no effect measured following albumin (Fig. 3F) or oleic acid treatment alone (Fig. 3G). Synergistic *de novo* phasic inhibition of TA nociceptive reflex activity across the SCI following albumin-oleic acid treatment was supported when compared to the presence of normal phasic facilitation following conditioning electrical stimulation in naïve animals without SCI from a peak of 1373±511% to 1764±173% (p<0.05, Fig. 3D).

**Figure 4:**
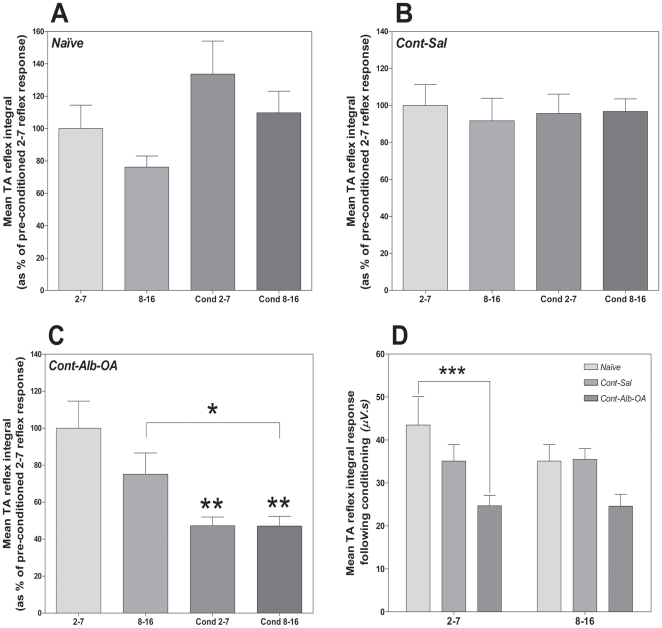
Albumin-Oleic Acid Promotes Phasic Inhibition of Early and Late Tibialis Anterior Reflex Activity following SCI. Analysis of the first and second phase of Tibialis Anterior (TA) reflex temporal summation in naïve animals (Fig. 4A) and those with SCI 28 days after T9 spinal contusion (Cont) revealed that the synergistic phasic inhibitory effect of Albumin-Oleic acid produced with the high-frequency electrical conditioning stimulation when normalized to the first phase of the pre-conditioned response was present during both the early and late phases of temporal summation (Fig. 4C), but not in the group treated with Saline (Sal, Fig. 4B) or in naïve animals (Fig. 4A). Furthermore activation of phasic inhibition of absolute integrated TA reflex activity measured during both phases of temporal summation revealed that Alb-OA treatment significantly inhibited nociceptive activity to below the normal value observed in naïve animals during the early phase (Fig. 4D). Statistical analysis was performed with a one-way ANOVA supported by a post-hoc Bonferroni test (* - p<0.05; ** - p<0.01, ***-p<0.001).

The synergistic *de novo* phasic inhibition of TA reflex activity following one month of albumin-oleic acid treatment was further supported when data were analyzed for both the early and late phase of temporal summation, where the second phase may provide indirect evidence of descending modulatory mechanisms (see methods, Fig. 4). Specifically albumin-oleic acid treatment inhibited the first phase from 100±15% to 47±5% (t = 3.43; d.f. = 20; p<0.01, Fig. 4C) and the second phase of TA nociceptive reflex temporal summation from 75±12% to 47±5% (t = 2.2; d.f. = 20; p<0.05, Fig. 4C). The significant decrease of the second phase of TA nociceptive reflex temporal summation suggested that albumin-oleic acid promoted phasic descending inhibitory control mechanisms (Fig. 4C). Furthermore *de novo* phasic inhibition of TA temporal summation with Alb-OA was shown to promote general antinociception when compared to noxious reflex activity measured in naïve animals (Fig. 4D).

### Potent synergistic increase in 5-HT innervation with Alb-OA after iSCI

Detailed analysis of the volume of T9 contusion SCI in all the experimental groups failed to identify general neuroprotection either with albumin, oleic acid or with the albumin-oleic acid complex. The volume of the contusion injury as a percentage of the total possible volume measured in naïve animals was 30.5±3.4% following SCI in the saline treated group. Similar values of SCI contusion volumes were measured with treatment with albumin alone (29.5±3.0%), oleic acid alone (28.9±2.1%), albumin-oleic acid (28.9±2.4%) and albumin-elaidic acid (33.3±3.1%).

**Figure 5:**
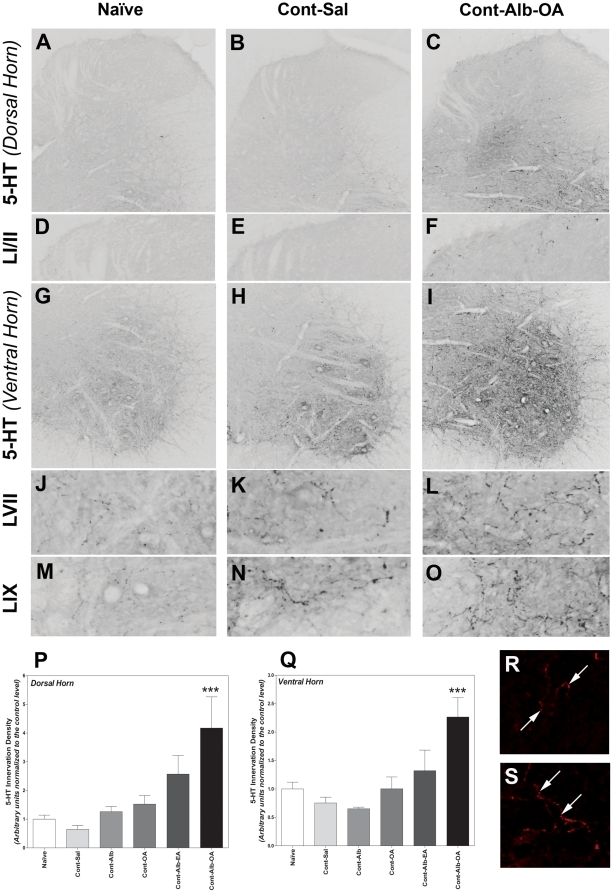
Albumin-Oleic Acid Increases Serotonin (5-HT) Innervation to Above Normal Levels after SCI. Immunohistochemical images revealed that 5-HT innervation density was increased within both the dorsal and ventral horn following Albumin-Oleic acid treatment (0.4/80 nanomoles, 5C and 5I, 10x) compared to either naïve animals (A and G, 10x) or those with SCI contusion (Cont) treated with saline alone (Sal, B and H, 10x). Detailed photographs of laminae I-II (D-F, note faint medial signal in D), lamina VII (J–L) and lamina IX (M–O) revealed the normal presence of 5-HT fibres with varicosities (D, J, M), following SCI (E, K, N) and in animals with spinal T9 contusion with albumin-oleic acid treatment (F, L, O). Quantification of the increase in lumbar 5-HT innervation levels below the SCI within the dorsal (P, p<0.001) or ventral horn (Q, p<0.001) following Albumin-OA treatment (Alb-OA, 0.4/80 nanomoles) was observed, but not after saline (Sal), albumin (Alb), oleic acid (OA) or albumin-elaidic acid (Alb-EA) administration. Statistical analysis was performed with a one-way ANOVA for each experimental group (P and Q, *** - p<0.001). Detailed original examples of fluorescent images of 5-HT fibres within laminae I–II (R) and laminae VII (S) are included with Alb-OA treatment 28 days after T9 SCI.

However immunohistochemical analysis revealed a potent increase in 5-HT innervation density below the T9 contusion SCI within the lumbar dorsal and ventral horn with albumin-oleic acid treatment. Although 5-HT innervation density was difficult to detect within the lumbar dorsal horn (Fig. 5A and 5B, see detailed photographs in 5D–5F), Alb-OA treatment revealed a notable generalized increase in lumbar dorsal horn 5-HT innervation density below the SCI (Fig. 5C and 5F). An upregulation of serotonin innervation with Alb-OA treatment was also evident in the ventral horn (Fig. 5G–5H) and in the detailed photographs of lamina VII (Fig. 5J–5L) and laminae IX (Fig. 5M–5O). Original microphotographs of 5-HT immunohistochemistry are included after Alb-OA treatment for lamina I–II (Fig. 5R) and lamina VII (Fig. 5S) at 28 days after SCI.

Quantification of serotoninergic innervation density measured below the SCI in animals treated with saline and normalised to the naïve level (Fig. 5P dorsal horn: 1.00±0.14, Fig. 5Q ventral horn: 1.00±0.12) revealed non-significant decreases of 5-HT levels within both the dorsal (0.65±0.14, Fig. 5P) and ventral horn (0.76±0.10, Fig. 5Q). In general significant differences were identified amongst all experimental groups within the dorsal (F = 6.79, d.f. = 5, p<0.001) and ventral horn (F = 6.96, d.f = 5, p<0.001). Only the albumin-oleic acid treatment significantly increased the normalised 5-HT innervation density levels to a remarkable 4.17±1.09 fold (p<0.001, Fig. 5P) within the dorsal horn and 2.27±0.34 fold (p<0.001, Fig. 5Q) within the ventral horn, when compared to animals treated with saline alone. Further evidence for a neurotrophic effect of albumin-oleic acid was evident by the significant difference between the observed increase in 5-HT innervation density compared to animals treated with albumin alone (dorsal horn: 1.26±0.18, p<0.01; ventral horn: 0.65±0.03, p<0.001) or with oleic acid alone (dorsal horn: 1.53±0.30, p<0.05; ventral horn: 1.00±0.21, p<0.05).

**Figure 6:**
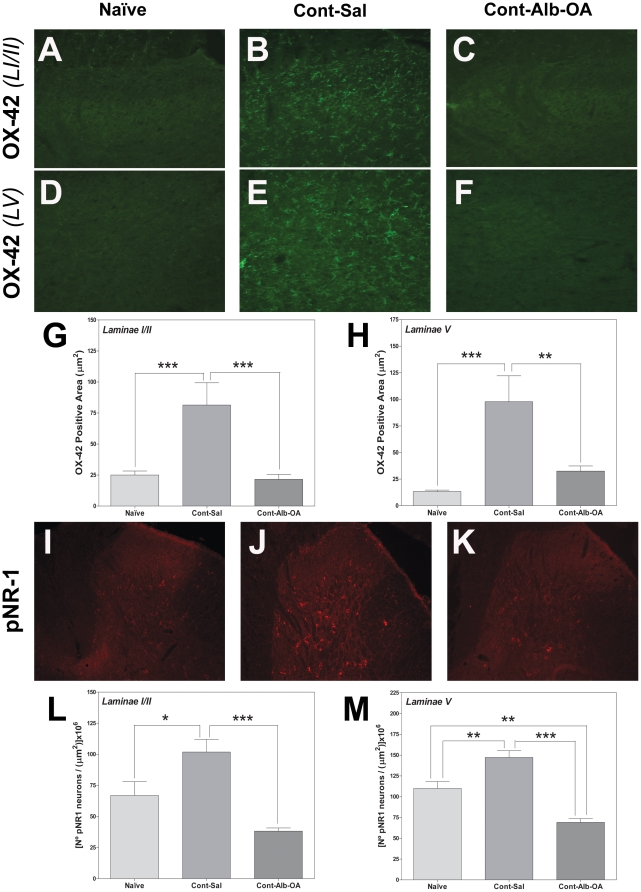
Albumin-Oleic Acid Decreases Microglia Activation and Reduces NMDA NR1 Phosphorylation after SCI. Alb-OA treatment (C and F, 20x) reduced microglia reactivity (OX-42, B and E, 20x) observed after SCI within both dorsal laminae I–II (C) and lamina V (F) when compared to naïve animals (A and D). Quantification of OX-42 area within laminae I–II (G) and lamina V (H) supported the qualitative inhibitory effect of Alb-OA treatment on increased microglia reactivity area following T9 contusion SCI when compared to naïve animals. Alb-OA treatment (K, 10x) reduced the accentuated number of cells with NR1 NMDA receptor phosphorylation (pNR-1) within the dorsal horn observed after SCI (J, 10x) when compared to normal tissue (I). Quantification of pNR1-positive cell number within the dorsal laminae I–II (L) and lamina V (M) supported the inhibition of NMDA receptor phosphorylation with Alb-OA. Statistical analysis was performed with an unpaired t-test (* - p<0.05; ** - p<0.01, *** - p<0.001).

The T9 spinal contusion injury increased microglia (OX-42) density within both the dorsal laminae I/II (Fig. 6B) and lamina V (Fig. 6E), accompanied by a generalized increase in the number of cells with phosphorylated NMDA NR1 receptor within the dorsal horn (Fig. 6J) compared to tissue from naïve animals (Fig. 6A, 6D, 6I). Importantly the albumin-oleic acid complex inhibited microglial OX-42 reactivity (Fig. 6C and 6F) and the number of pNR1-positive cell counts within the dorsal horn to those observed in naïve animals (Fig. 6K).

Spinal T9 contusion injury led to an increase in microglial OX-42 area within the superficial (LI/II: naïve 25.1±3.2, contusion 81.5±0.18, p<0.001, Fig. 6G) and deep dorsal horn (LV: naïve 13.3±1.4, contusion 97.8±24.3, p<0.001, Fig. 6H). Compared to microglial reactivity following SCI, the OX-42 area decreased within both the superficial (21.6±3.9, p<0.001, Fig. 6G) and deep dorsal horn (32.6±4.80, p<0.01, Fig. 6H) following albumin-oleic acid treatment.

When compared to naïve animals, an increase in the number of pNR1-positive cells following SCI was observed within the superficial dorsal horn from 66.9±11.1 to 101.9±10.1 (p<0.05, Fig. 6L) and deep dorsal horn 110.0±8.3 to 147.3±8.1 (p<0.01, Fig. 6M). Treatment with albumin-oleic acid reduced the number of pNR1-positive cells compared to the SCI group treated with saline alone within the superficial (38.2±2.6, p<0.001, Fig. 6L) and deeper dorsal horn (69.2±4.5, p<0.001, Fig. 6M). Interestingly within the deep dorsal horn, albumin-oleic acid treatment decreased pNR1-positive cells to below that identified in naïve levels (69.2±4.5 vs. 110.0±8.3, p<0.01, Fig. 6M), suggesting a clear antinociceptive effect.

## Discussion

The novel effect of intrathecal albumin, oleic acid and especially Alb-OA treatment on spinal and descending inhibition of TA nociceptive reflex activity and early recovery of voluntary motor function following iSCI is described for the first time in this study, and is supported by the simultaneous modulation of three spinal cellular mechanisms at the lumbar level. These include reduction of NR1 NMDA receptor phosphorylation, inhibition of microglial reactivity and a strong increase in 5-HT levels to above normal levels within the dorsal and ventral horn. The specific synergistic effect of the putative neurotrophic factor Alb-OA on promotion of tonic and *de novo* phasic descending inhibition of spinal noxious reflex excitability across the iSCI is notable. In addition the measurement of Tibialis Anterior reflex activity using standard spinal and a newly developed translational measure of descending function assures the clinical relevance of the effect of Alb-OA as a novel neurotrophic factor *in vivo* following central neurotrauma. The potentially safe clinical profile of this complex [50] and to the possibility that specific SCI symptoms of spinal change in nociceptive function and spasticity could be treated with Alb-OA is discussed below.

### Pleiotropic effect of Alb-OA on noxious central sensitisation and paralysis after iSCI

Albumin comprises of about half of blood serum protein and has multiple specific binding sites for fatty acids [23], [51]. Motor recovery and neuroprotection have been demonstrated with both albumin [21] and omega 3 fatty acids following iSCI [22], [49]. Although OA is a known neurotrophic factor *in vitro* [20], a functional effect of systemic administration of this omega-9 fatty acid has not been demonstrated alone [22], and is supported by the poor motor recovery following SCI observed in our study. In contrast inhibition of flexor reflex activity was identified individually with either Alb or OA, as predicted by other studies [21], [52]–[54]. Surprisingly synergistic early motor recovery and *de novo* phasic inhibition of spinal nociceptive reflex activity was observed only when both molecules were combined, which suggests that the neurotrophic effect of this complex *in vivo* is likely to be mediated via collateral sprouting of residual descending modulatory pathways after iSCI [37]. An *in vitro* study demonstrates that extracellular Alb and OA are incorporated into neurons leading to dendritic growth and GAP-43 upregulation [20]. At the *in vivo* level CNS injury leads to SCD-1 upregulation, the enzyme responsible for oleic acid synthesis [55], which is also related to normal myelin production [56]. Another *in vitro* study has demonstrated that oleic acid-mediated upregulation of GAP-43 is synergistic with the neurotrophic factors NT3 and NT4/5 [57]. Interestingly the role of these factors have been related to the prevention of corticospinal tract atrophy [58] and the promotion of serotoninergic innervation below iSCI [59].

The application of the non-invasive electrical conditioning protocol developed to indirectly assess the role of descending pathways across the iSCI was adapted from Liu et al. (1998), which examined long-term depression (LTD) of C-fibre-evoked field potentials [47]. Interestingly in the original study LTD was shown to convert to long-term potentiation when the spinal cord was transected, suggesting that the activation of descending inhibitory control systems maybe critical for the observation of spinal reflex inhibition *in vivo* [47]. Although phasic descending of noxious reflex excitability has been demonstrated in uninjured animals with the application of other electrical conditioning stimuli remote to the test site [34], this is the first time that this technique has revealed descending modulatory system plasticity following iSCI following treatment, although the role of altered propriospinal system function through the iSCI cannot be excluded [60], [61].

### Alb-OA modulates iSCI neuronal excitability, neuroinflammation and serotonin neuroplasticity

Albumin-OA mediated a strong increase in serotonin innervation density within both the dorsal and ventral lumbar level, higher than that observed following other experimental treatments [37], [62] or indeed to that found in uninjured spinal tissue. Oleic acid is an allosteric factor for the 5-HT7_A_ receptor [63], and this receptor is present within the superficial dorsal horn [64] and may play a role in analgesia [65]. As such tonic inhibition of nociceptive TA reflex temporal summation could be mediated via 5-HT7_A_ receptor activation [66]. Although there are many 5-HT receptor subtypes with mixed inhibitory and facilitatory neuronal effects, enhanced neuroplasticity of residual descending systems with a phenotypic shift from serotoninergic facilitation to inhibition of spinal function [66] would be expected to overcome 5-HT supersensitivity which has been implicated as a pathophysiological mechanism of change in nociception [18] and motor function [17] following SCI.

At the lumbar spinal level inhibition of nociceptive TA reflex temporal summation with Alb-OA was reflected also by a decrease in the number of lumbar dorsal horn neurons with phosphorylated NR1 receptors. The NR1 NMDA receptor subtype is present within the dorsal horn [67] and on motoneurones [68], and specific variants of this receptor are upregulated after contusion SCI [69]. Temporal summation of nociceptive flexor reflex activity can be pharmacologically blocked with NMDA antagonists following ischemic SCI [70], and NR1 phosphorylation is related to central spinal sensitisation to noxious stimuli [71]. Importantly the relationship between descending inhibitory systems and reduction of pNR1 activation has been demonstrated recently with the application of monoaminergic agonists following NMDA administration [72], suggesting the hypothesis that increased descending serotoninergic tract function promoted by Alb-OA treatment could mediate a reduction in nociceptive reflex excitability via control of the pNR1 NMDA receptor subtype below the iSCI.

Albumin-OA treatment also strongly reduced microglia reactivity within the lumbar dorsal horn following SCI. Microglia activation has been identified up to several weeks following SCI in humans [73] and is part of the normal inflammatory response [74]. Furthermore microglia reactivity has been implicated in central sensitisation to noxious stimuli [16] and in patients following SCI with symptoms of spasticity [75]. Oleic acid inhibits the induction of proinflammatory mediators, such as NF-κB, within microglial cell culture [54], and so may have a direct effect against neuroinflammation. In parallel with the hypothesis suggested above for the interaction between descending serotoninergic and spinal mechanisms of excitability modulated via the pNR1 NMDA receptor, the application of noradrenergic agonists such as clonidine has been shown to inhibit GFAP upregulation, nuclear factor-kappa B activation, and p38 mitogen-activated protein kinase induction following peripheral nerve injury [76]. As such Albumin-OA complex treatment may control spinal excitability following iSCI following descending 5-HT inhibitory tract neuroplasticity, which in turn would inhibit microglia reactivity and NMDA receptor phosphorylation following iSCI.

### Clinical significance of Alb-OA treatment for iSCI and remaining key experiments

Simultaneous successful treatment of SCI central sensitisation to noxious stimuli, spasticity, and paralysis below the injury depends on the control of several spinal pathophysiological mechanisms [2], [77]–[80], which can be broadly grouped into processes of neuronal excitability, neuroinflammation and plasticity of sensorimotor circuits following partial or complete damage to the spinal cord [7]–[10]. Novel pleiotropic treatments are not only required to modulate these basic mechanisms [9], [11]–[13], but to target the most debilitating symptoms of spasticity and pain [2], [81], [82], without precipitating secondary effects of somnolence or fatigue [83]–[85]. Neurophysiological evidence for the modulation of non-invasive translational measures of spinal nociceptive reflex activity such as those used here, combined with immunohistochemical evidence for the role of three cellular mechanisms of action related to the control of spinal sensorimotor function, demonstrates that the Alb-OA complex meets all of these criterion.

While Alb has a an established clinical safety profile [86], only indirect evidence exists for OA as a non-toxic agent according to one nutritional study of olive oil administration [50]. Importantly in our study no change in feeding behaviour, body weight or grooming was observed with Alb-OA treatment following iSCI. Albumin most probably provides therapeutic benefits for spinal cord injury when administered alone [21]. Indeed the replacement of cerebrospinal fluid with 8% albumin following experimental ischaemic experimental SCI can reduce motor deficits, although this has been attributed mainly to reduction of intraspinal oedema [87]. However evidence for direct penetrability of Alb-OA into the spinal parenchyma should be addressed in future studies. Functional motor recovery has been identified when Alb is injected directly into the spinal tissue or via the intravenous route [21], but careful analysis should be performed with FITC-albumin injected via the intrathecal route which would address this concern. Indeed a similar study has identified both grey and white spinal matter labelling with FITC-albumin when administered via the intravenous route, confirming penetration at least into the intraspinal microcirculatory system [87]. Albumin is commonly used a drug delivery system in the clinical setting [88] and specific known binding sites with OA suggest a biological basis for the synergistic action between both molecules observed in this study [89]. As such drug delivery of OA across the blood brain barrier could be mediated by albumin transcytosis [90], [91], followed by cellular uptake within the spinal parenchyma [19], and synergistic modulation of sensorimotor function following iSCI.

Of course several limitations regarding the preclinical development and translational application of Alb-OA still remain, including the possibility of its administration via the systemic route and characterisation of the therapeutic window. General neuroprotection following SCI was not observed with Alb-OA in this study, in direct contrast to the effect of omega-3 fatty acids such as docosahexanoic acid alone [22], or in combination with albumin in a stroke model [92]. The neurotrophic effect of Albumin-OA *in vivo* also needs to be addressed directly through pharmacological blockade of the peroxisome proliferator activated receptors, the role of which have been highlighted in an *in vitro* study [93]. Finally, further evidence for specific cellular *in vivo* mechanisms of action of Alb-OA should now be extended with invasive neuroanatomical and neurophysiological techniques to further understand the role of Alb-OA on both extrapyramidal and corticospinal descending control systems across the iSCI.

### Conclusion

The Albumin-Oleic acid complex is a new pharmacological treatment strategy with pleiotropic functional effects for the symptoms of paralysis, spasticity and change in spinal pain processing following iSCI. Initial evidence of specific mechanisms of action include the simultaneous tonic and *de novo* phasic descending inhibition of spinal reflex excitability mediated by a potent increase in lumbar 5-HT innervation, with a reduction in NR1 NMDA receptor phosphorylation and microglial reactivity. Neurotrophic enhancement of collateral sprouting of residual 5-HT fibres across the iSCI has been demonstrated to mediate locomotor recovery [38], [39]. However the effective inhibition by Alb-OA of a translational non-invasive measure of the most debilitating symptoms of spasticity and change in spinal nociception [81], [82], while promoting residual voluntary motor function below the lesion, represents an important clinical goal for subacute rehabilitation of patients with iSCI.
